# The relationship between first trimester maternal diet and early pregnancy loss: a retrospective case–control study

**DOI:** 10.1186/s12884-026-08721-1

**Published:** 2026-02-07

**Authors:** Muhammed Bartu Varol, Berkin Özyilmaz Kircali

**Affiliations:** 1 Department of Nutrition and Dietetics, Faculty of Health Sciences, Istanbul Yeni Yuzyil University, Istanbul, 34010 Turkey; 2 Deparment of Nutrition and Dietetics, Graduate Education Institute, Istanbul Aydin University, Istanbul, 34295 Turkey; 3 Department of Nutrition and Dietetics, Faculty of Health Sciences, Istanbul Aydin University, Istanbul, 34295 Turkey

**Keywords:** Maternal diet, Early pregnancy loss, Dietary fiber

## Abstract

**Background:**

Early pregnancy loss (EPL) is a global public health concern with significant physical and psychological effects on individuals and society. The specific etiology of many EPL cases is not well understood, and first trimester maternal diet may play a role in EPL occurrence. This study aimed to examine the differences in first trimester maternal nutrient intake, nutritional habits and nutritional knowledge levels between healthy pregnant women and women who experienced EPL.

**Methods:**

A single center retrospective case–control study was conducted at a public hospital in Istanbul, Turkey between May–October 2024. The case group comprised 65 women who experienced first-time pregnancy loss. The control group included 65 healthy pregnant women in the first trimester without a previous PL history, with both groups matched for age, body mass index (BMI) and parity. Data were collected from the participants regarding their general information, dietary habits, the Quantitative Food Frequency Questionnaire (QFFQ), and the Nutrition Knowledge Level Scale for Adults (NKLSA). Dietary nutrient intakes were evaluated on the basis of the Dietary Reference Intakes (DRI) values and compared between the two groups. To further investigate the link between specific dietary nutrient intakes and EPL risk, adjusted binary logistic regression models were employed.

**Results:**

The mean age of the participants was 28.5 years, and their mean BMI was 24.20 kg/m^2^. The dietary intake of carbohydrates, dietary fiber, monounsaturated fatty acids, omega-3 fatty acids, folate, vitamin C, potassium, iodine and total minerals were statistically higher in the control group (*p* < 0.05). Dietary vitamin D and cholesterol intake and the omega-6/omega-3 ratio were greater in the case group without statistical significance (*p* > 0.05). The control group presented significantly higher proportions of dietary supplement use, regular consumption between meals, and higher daily meal number (*p* < 0.05). Adjusted binary logistic regression analysis revealed negative correlations between total dietary fiber intake (continuous and Q3/Q4) and EPL risk in all models, even after adjusting for all potential confounders (*p* < 0.05). There was no statistically difference regarding nutrition knowledge level between two groups (*p* > 0.05).

**Conclusions:**

This study contributes to the literature by showing the protective role of maternal nutritional status in the first trimester against EPL risk.

**Graphical Abstract:**

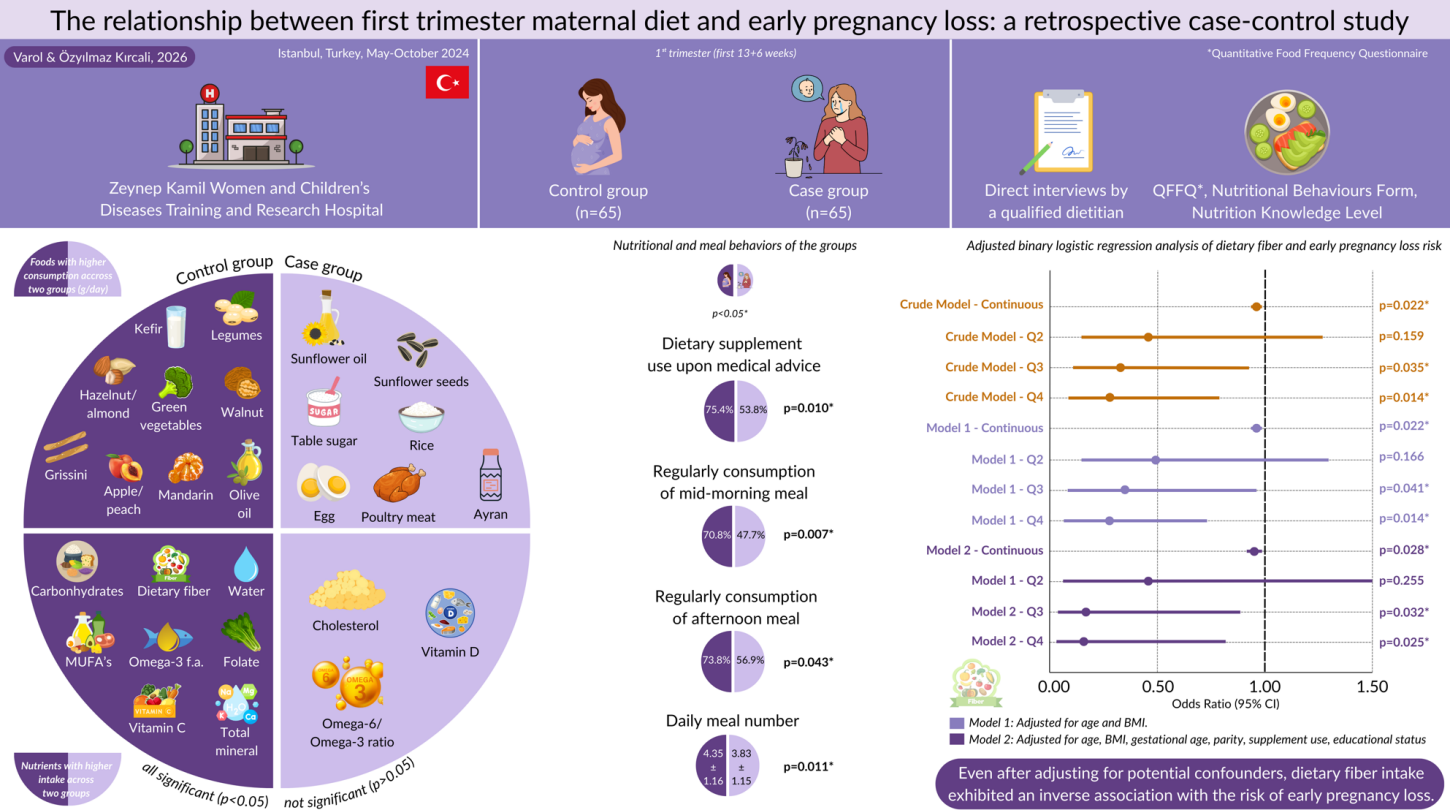

## Introduction

Pregnancy loss (PL) is the expulsion of an embryo or fetus from the uterus during the first 20 gestational weeks weighing 500 g or less, without any signs of life [1]. PL represents a serious public health issue associated with parental psychological disorders and overall health impairment, which may further influence the outcomes of future pregnancies [2]. PL is one of the most common adverse outcomes of pregnancy, accounting for 10–15% of all monitored pregnancies [3]. Approximately 23 million PL cases are reported worldwide annually, corresponding to about 44 cases per minute [4]. According to the Turkey Demographic and Health Survey (2018), one in five married women in Turkey experience PL during their lifetime [5]. Early pregnancy loss (EPL) is defined as the occurrence of PL within the first trimester and constitutes nearly 80% of all PL cases [6].

It is widely accepted that 50% of all PLs are caused by chromosomal abnormalities [7]. However, the cause of 50% of miscarriages cases has not been fully identified, and it is estimated that various factors may contribute to PL [8]. Advanced maternal age [9] and prior history of PL [10] have been recognized as the most predictive risk factors for PL. High parity [11] and gravidity [12], diabetes mellitus (DM) [3], thyroid dysfunction [13], chronic kidney disease [14] and hypertension [15] are other risk factors for PL. Lifestyle factors such as smoking [16], alcohol consumption [17], increased caffeine intake [18] and the use of certain medications [19] also may play a role in PL.

Maternal nutritional status appears to have great potential in the prevention of PL [20]. An optimal diet beginning with the preconception period is essential for maternal homeostasis, fetal growth and healthy pregnancy progression [21–23]. Micronutrients, such as vitamins and minerals, are reported to reduce the risk of PL by contributing to embryogenesis and placental development [24]. Unbalanced maternal nutrition, underweight and obesity contribute to fetal abnormalities and negative pregnancy outcomes including PL [25]. Little is known about the differences in nutritional status between healthy pregnant women and women who have EPL.

This study aimed to examine the potential effects of first trimester maternal nutrient intake, nutritional habits and nutritional knowledge levels on EPL while controlling for other risk factors.

## Materials and methods

### Study design and population

A single center retrospective case–control study was undertaken between May and October 2024 in the Istanbul Zeynep Kamil Women's and Children's Diseases Training and Research Hospital, Turkey. The case group comprised 65 women who were diagnosed with EPL during first trimester and referred to the septic unit for curettage surgery by a doctor. The control group included 65 healthy pregnant women who attended routine antenatal visits in the first trimester and were matched for age, BMI and parity. The first trimester was considered the first 13 + 6 weeks of gestation, according to the definitions of the The American College of Obstetricians and Gynecologists (ACOG) and the International Classification of Diseases, Tenth Revision (ICD-10) [26].

### Inclusion and exclusion criteria

The inclusion criteria for both groups were healthy women aged 18–35 years with a BMI between 18.50–29.99 kg/m^2^ and a naturally conceived singleton pregnancy.

**Fig. 1:**
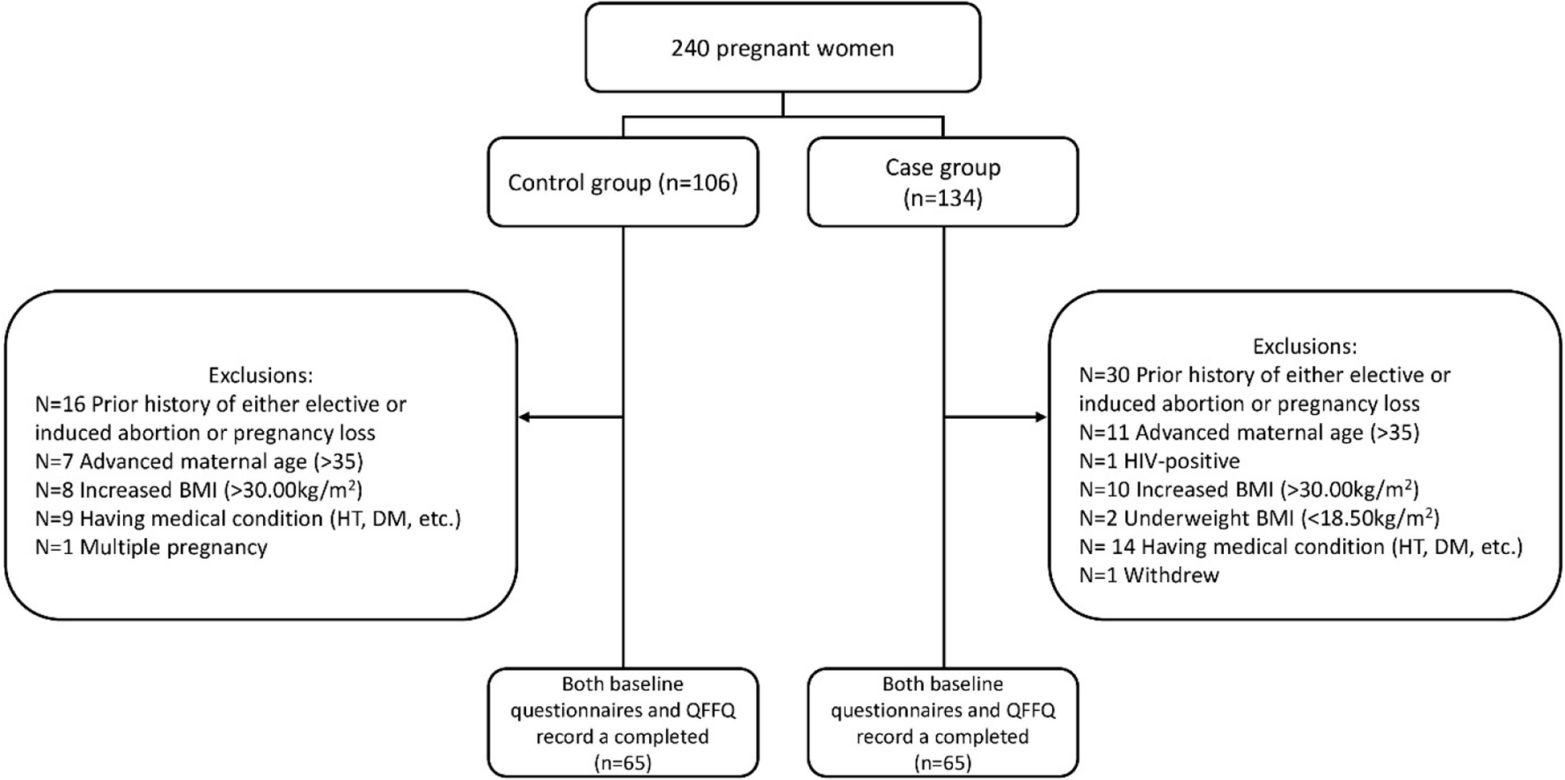
Flowchart of study population

The exclusion criteria were multiple pregnancies, conceived by assisted reproductive technologies, a prior history of either elective/induced abortion or EPL, and preexisting medical conditions including DM, chronic kidney disease, thyroid disease, chronic hypertension, or HIV infection (Fig. 1).

### Data collection

Women attending Istanbul Zeynep Kamil Women's and Children's Diseases Training and Research Hospital in Turkey between May–October 2024 were screened for eligibility. Those meeting the inclusion criteria were recruited using a convenience sampling method by a qualified dietitian via face-to-face interviews. Specifically, the control group was recruited during routine antenatal visits, while the case group was enrolled before the curettage procedure in the septic unit of the same hospital. Clarify that participation was entirely voluntary; therefore, written informed consent form were obtained from all participants. The following data were collected: general characteristics (Table 1) and, nutritional and meal behaviors forms (Table 2), the Nutrition Knowledge Level Scale for Adults (NKLSA) and quantitative food frequency questionnaire (QFFQ).

The NKLSA was developed and validated by Batmaz, H. (2018), was applied after the necessary permission obtained for its use [27]. The NKLSA, consists of two Likert-type scales [1-Basic nutrition (score classified as low: < 45p, moderate: 45–55p, high: 56–65p, very high: > 65p), 2-Food choices (score classified as low: < 30p, moderate: 30–36p, high: 37–42p, very high: > 42p)], and two 0–10 point visual analog scales (VASs) [1-Subjective assessment of the nutrition-health relationship (*What is the degree of the relationship between nutrition and health?*), 2-Self-administered healthy food preferences (*How appropriate do you consider your dietary choices in daily life?*)]. The scale demonstrated that Cronbach’s alpha coefficients of 0.72 for the “Basic nutrition” section and 0.70 for the “Food choices” section.

The dietary nutrient intake of the participants was assessed using a QFFQ consisting of thirty-seven food items from five different food groups (1-dairy products; 2-meat, legumes and nuts; 3-bread and cereals; 4-fruits and vegetables; 5-fats and sugars). The frequency categories were adopted from a validated Food Frequency Questionnaire (FFQ) developed by Satia et al. (2009) [28]. These categories were converted into daily numerical factors using the standard class mid-point method [29]. A conservative scoring approach was applied to minimize overestimation. The following daily conversion factors were used: never in the past month; 0, once a month; 0.033, two or three times a month; 0.067, one or two times a week; 0.2, three or four times a week; 0.5, five or six times a week; 0.7, once a day; 1.0, two or more times a day; 2.0. The standard portion sizes in Turkey were obtained from 2022 Turkey Dietary Guidelines (TUBER) [30], and images of standard portions was used to determine the exact serving size. The portion size categories for all food items and their conversion factors were as follows: small portion; 0.5, medium portion; 1.0, large portion; 1.5 [31]. The daily consumption of each food item was calculated by multiplying the conversion factors by both the frequency and portion size. The participants’ daily energy and nutrient intakes were calculated by entering the data into BeBiS software (Nutrition Information Systems, version 7.2, Istanbul, Turkey). Total daily dietary macro- and micronutrient intakes were evaluated on the basis of the recommended dietary allowance (RDA) and adequate intake (AI) values for pregnant women aged 19–30 years and classified as insufficient (< 50%), acceptable (50–100%) or sufficient (> 100%) [32–34].

### Statistical analysis

Power analysis was performed via G*Power software (version 3.1.9.6) to determine the sample size. To obtain 80% power at the α = 0.05 level, at least 64 participants were included in each group in this study. A total of 240 individuals were screened, and the study was conducted with 130 participants (65 participants from each group). The normality of continuous variables was tested on the basis of skewness and kurtosis values, with values between −1.50 and + 1.50 considered to indicate a normal distribution [35]. Variables with a normal distribution were compared between two groups via the independent samples t-test. Non-normally distributed variables compared using the Mann–Whitney U test. Differences between categorical variables were examined via the Pearson Chi-Square test or Fisher’s exact test when assumptions were not met. Descriptive statistics are presented as the means with standard deviations for continuous variables and frequencies with percentages for categorical variables. Adjusted binary logistic regression was employed to investigate the association between dietary intake of specific nutrient, with odds ratios (ORs) and 95% confidence intervals (CIs) calculated for each variable. The dietary intake of these nutrients was included in the models for both continuous and categorical variables (Q1–Q4), with the lowest quartile (Q1) serving as the reference group. The crude model did not adjust for any covariates, whereas Model 1 accounted for age and BMI. Model 2 was additionally adjusted for gestational age, parity, supplement use and educational status. A *p*-value < 0.05 was considered to indicate statistical significance.

All the statistical analyses were performed via the IBM SPSS Statistics, version 26.0 (IBM Corp., Armonk, NY, USA).

### Ethical approval

This study was approved by the Non-Interventional Clinical Research Ethics Committee of Istanbul Aydin University (Protocol no.: 41/2024). Before the data collection, written permission was also obtained from the hospital administration where the study was conducted.

## Results

The general characteristics of the study participants are shown in Table 1. Maternal age, BMI, parity, medical condition, prescription medication use, educational and employment status, smoking and alcohol use, and daily walking time variables were similar between the case and control groups (*p* > 0.05). However, significant differences were noted in terms of gestational age (*p* < 0.001).

**Table 1:** General characteristics of the groups

Variables	Total (*n* = 130)	Control group (*n* = 65)	Case group (*n* = 65)	p
Age (years)^†^	28.50 ± 4.30	28.60 ± 4.20	28.40 ± 4.30	0.728
Height (m)^†^	1.63 ± 0.06	1.63 ± 0.06	1.63 ± 0.07	0.755
Weight (kg)^†^	64.20 ± 10.60	64.00 ± 9.80	64.40 ± 11.30	0.826
BMI (kg/m^2^)^†^	24.20 ± 3.30	24.10 ± 3.20	24.30 ± 3.50	0.682
Gestational age (week)^‡^	10.60 ± 2.40	12.17 ± 1.59	9.08 ± 2.03	0.000*
Parity (n)^‡^	0.60 ± 0.90	0.57 ± 0.90	0.63 ± 0.89	0.686
Medical condition (Yes, n, %)^a^	11 (8.5)	6 (9.2)	5 (7.7)	0.753
Prescription medication use (Yes, n, %)^b^	8 (6.2)	6 (9.2)	2 (3.1)	0.273
Educational status (associate degree or higher, n, %)	61 (46.9)	40 (61.5)	29 (44.6)	0.053
Employment (Yes, n, %)	41 (31.5)	21 (32.3)	20 (30.8)	0.850
Smoking during pregnancy (Yes, n, %)	24 (18.5)	12 (18.5)	12 (18.5)	1.000
Alcohol consumption during pregnancy (Yes, n, %)	1 (0.8)	0 (0.0)	1 (1.5)	1.000
Daily walking time (min/day)^‡^	34.10 ± 32.40	33.75 ± 31.33	34.52 ± 33.71	0.963

^†^Independent samples t-test, ^‡^Mann–Whitney U test, *p* < 0.05*

^a^Medical conditions in the control group consisted of seasonal allergies (*n* = 5) and ulcerative colitis (*n* = 1), whereas the case group included seasonal allergies (*n* = 4) and vertigo (*n* = 1)

^b^Prescription medications in the control group received antihistamines (*n* = 5) and intestinal anti-inflammatory agents (*n* = 1), while the case group included antihistamines (*n* = 1) and antivertigo agents (*n* = 1)

Nutritional behaviors, summarized in Table 2, were significantly different between the two groups. Dietary supplement use upon medical advice was significantly higher in the control group than in the case group (75.4% vs. 53.8%, *p* = 0.010). Regular consumption of mid-morning and afternoon meals were significantly higher in control group (70.8% vs. 47.7%, *p* = 0.07 and 73.8% vs. 56.9%, *p* = 0.043, respectively). However, regular consumption of late-night meal was higher in the case group without statistical significance (46.2% vs. 33.8%, *p* > 0.05). The control group presented a significantly higher daily meal number (4.35 ± 1.16 vs. 3.83 ± 1.15, *p* = 0.011). In both groups, the most frequently skipped meals were lunch (48.9% control, 54.2% case) and breakfast (17.8% control, 27.1% case). The proportion of participants who received nutritional counseling before or during pregnancy was only 18.5% in the control group and 15.4% in the case group (*p* > 0.05).

**Table 2:** Nutritional and meal behaviors of the groups

Variables	Total (*n* = 130)	Control group (*n* = 65)	Case group (*n* = 65)	p
Water consumption (ml/day)^‡^	1660.00 ± 908.75	1710.77 ± 1008.73	1609.23 ± 801.12	0.776
Dietary supplement use upon medical advice (Yes, n, %)	84 (64.6)	49 (75.4)	35 (53.8)	0.010*
*Regular consumed meals (Yes, n, %)*				
Breakfast	112 (86.2)	59 (90.8)	53 (81.5)	0.128
Mid-morning	77 (59.2)	46 (70.8)	31 (47.7)	0.007*
Lunch	83 (63.9)	45 (69.2)	38 (58.5)	0.201
Afternoon	85 (65.4)	48 (73.8)	37 (56.9)	0.043*
Dinner	122 (93.8)	62 (95.4)	60 (92.3)	0.718
Late-night	52 (40.0)	22 (33.8)	30 (46.2)	0.152
Regular skipping any meals (Yes, n, %)	93 (71.5)	45 (69.2)	48 (73.8)	0.560
*Most frequently skipped meal (n, %)*				
Breakfast	21 (22.6)	8 (17.8)	13 (27.1)	0.554
Mid-morning	7 (7.5)	5 (11.1)	2 (4.1)	
Lunch	48 (51.6)	22 (48.9)	26 (54.2)	
Afternoon	7 (7.5)	5 (11.1)	2 (4.1)	
Dinner	7 (7.5)	3 (6.7)	4 (8.3)	
Late-night	3 (3.2)	2 (4.4)	1 (2.1)	
Daily meal number (n)^†^	4.10 ± 1.18	4.35 ± 1.16	3.83 ± 1.15	0.011*
Nutritional counselling before or during pregnancy (Yes, n, %)	22 (17.0)	12 (18.5)	10 (15.4)	0.640
Nutritional counselling from dietitian (Yes, n, %)	16 (12.3)	10 (15.4)	6 (9.2)	0.286

^†^Independent samples t-test, ^‡^Mann–Whitney U test, *p* < 0.05*

The NKLSA scores of both groups are presented in Table 3. The “Basic nutrition”, “Food choices” and “self-administered healthy food preferences” scores were similar between the two groups (*p* > 0.05). The control group presented significantly higher “subjective assessment of the nutrition-health relationship” VAS scores (8.51 ± 1.80 vs. 7.55 ± 2.56, *p* = 0.015).

**Table 3:** NKLSA scores of the participants

Variables	Control group (*n* = 65)	Case group (*n* = 65)	p
Basic nutrition^†^	52.17 ± 7.09	51.35 ± 6.15	0.485
Food choices^†^	37.26 ± 5.79	36.45 ± 5.59	0.416
Subjective assessment of the nutrition-health relationship (scale: 0–10 points)^†^	8.51 ± 1.80	7.55 ± 2.56	0.015*
Self-administered healthy food preferences (scale: 0–10 points)^†^	5.69 ± 1.67	5.25 ± 2.00	0.170

^†^Independent samples t-test, *p* < 0.05*

The food consumption frequencies and daily amounts of the participants are given in Table 4. The control group had a significantly greater consumption frequency and daily intake (g/day) of kefir, legumes, hazelnut/almond, walnut, grissini, apple/peach, mandarin and olive oil (*p* < 0.05). The case group had higher consumption frequency and daily intake (g/day) of egg, poultry meat, rice, sunflower seeds and sunflower oil without statistical significance (*p* > 0.05).

**Table 4:** Consumption frequencies and daily amounts of food items derived from the QFFQ

	Consumption frequencies			Daily amounts (g)		
Variables	Control group (*n* = 65)	Case group (*n* = 65)	p	Control group (*n* = 65)	Case group (*n* = 65)	p
Milk	4.08 ± 2.17	3.63 ± 2.10	0.236^†^	95.01 ± 107.43	74.10 ± 96.24	0.242^‡^
Yogurt	5.85 ± 1.44	5.49 ± 1.54	0.179^†^	177.23 ± 127.43	159.03 ± 127.64	0.439^‡^
Kefir	2.62 ± 2.07	1.85 ± 1.62	0.016^‡^*	49.35 ± 109.60	21.36 ± 63.72	0.016^‡^*
Ayran	4.80 ± 1.66	4.72 ± 1.82	0.801^†^	175.09 ± 170.03	179.04 ± 235.01	0.343^‡^
White cheese	6.22 ± 1.43	5.74 ± 1.87	0.188^‡^	52.55 ± 42.87	45.12 ± 32.76	0.578^‡^
Kashar cheese	5.00 ± 1.94	4.80 ± 2.09	0.573^†^	22.82 ± 24.29	22.65 ± 22.14	0.920^‡^
Egg	5.52 ± 1.72	5.71 ± 1.52	0.518^†^	44.56 ± 41.60	50.77 ± 44.47	0.562^‡^
Red meat	3.69 ± 1.29	3.52 ± 1.31	0.459^†^	16.87 ± 15.58	16.02 ± 17.34	0.438^‡^
Poultry meat	3.74 ± 1.38	4.02 ± 1.05	0.202^†^	19.73 ± 19.61	24.21 ± 21.70	0.220^†^
Fish	2.45 ± 1.29	2.09 ± 1.21	0.105^‡^	14.65 ± 19.91	11.87 ± 30.15	0.206^‡^
Legumes	3.58 ± 1.13	2.98 ± 1.37	0.008^†^*	25.07 ± 27.63	18.40 ± 28.54	0.015^‡*^
Hazelnut/almond	5.43 ± 1.53	4.25 ± 1.90	0.000^†^*	12.18 ± 10.52	8.61 ± 13.81	0.001^‡*^
Walnut	5.38 ± 1.67	4.02 ± 2.01	0.000^†^*	13.99 ± 13.97	8.75 ± 13.41	0.000^‡^*
Sunflower seeds	3.22 ± 2.06	3.45 ± 2.09	0.527^†^	13.49 ± 28.13	17.29 ± 30.53	0.400^‡^
Bread	6.66 ± 1.36	6.26 ± 1.65	0.188^‡^	64.24 ± 46.51	58.99 ± 44.74	0.513^†^
Turkish sesame bagel	3.37 ± 1.38	3.17 ± 1.52	0.432^†^	11.14 ± 14.77	10.39 ± 15.30	0.396^‡^
Soup	5.65 ± 1.37	4.95 ± 1.85	0.017^†^*	132.33 ± 104.42	110.08 ± 98.85	0.134^‡^
Rice	4.15 ± 1.19	4.20 ± 1.37	0.838^†^	30.35 ± 29.57	33.97 ± 34.27	0.769^‡^
Bulgur	4.02 ± 1.29	3.68 ± 1.37	0.150^†^	30.33 ± 39.54	22.36 ± 24.24	0.192^‡^
Pasta	4.02 ± 1.30	3.72 ± 1.39	0.218^†^	26.75 ± 35.53	23.10 ± 26.18	0.354^‡^
Grissini	2.25 ± 1.68	1.51 ± 1.05	0.002^‡^*	3.40 ± 8.11	1.01 ± 3.00	0.003^‡^*
Corn flakes	1.66 ± 1.46	1.51 ± 1.17	0.661^‡^	2.46 ± 8.62	1.01 ± 4.03	0.664^‡^
Dark leafy greens (spinach/lettuce)	4.60 ± 1.46	4.42 ± 1.52	0.481^†^	94.21 ± 80.22	85.22 ± 89.98	0.244^‡^
Green vegetables (cucumber/green beans/peas/broccoli)	5.32 ± 1.30	4.91 ± 1.55	0.100^†^	116.31 ± 94.37	91.77 ± 96.66	0.021^‡^*
Red vegetables (tomato/carrot/red pepper/eggplant)	5.49 ± 1.42	5.40 ± 1.41	0.710^†^	122.14 ± 89.20	110.57 ± 83.69	0.406^‡^
Potato	4.85 ± 1.30	4.66 ± 1.28	0.416^†^	56.41 ± 48.34	49.95 ± 40.13	0.526^‡^
Apple/peach	5.68 ± 1.61	4.40 ± 1.56	0.000^†^*	90.46 ± 71.26	48.77 ± 46.61	0.000^‡^*
Mandarin	3.58 ± 2.46	2.55 ± 1.98	0.009^†^*	88.59 ± 143.03	41.86 ± 77.43	0.022^‡^*
Dried fruits	3.83 ± 2.23	3.49 ± 2.10	0.375^†^	9.53 ± 10.54	9.05 ± 15.05	0.456^‡^
Olive oil	5.17 ± 2.30	4.09 ± 2.60	0.014^†^*	4.57 ± 4.93	3.33 ± 4.35	0.034^‡^*
Olive	6.11 ± 1.51	5.63 ± 1.88	0.189^‡^	7.28 ± 6.10	5.64 ± 3.90	0.141^‡^
Sunflower oil	5.46 ± 1.96	5.60 ± 2.06	0.695^†^	4.12 ± 3.54	4.91 ± 4.56	0.640^‡^
Butter	3.83 ± 2.23	3.37 ± 2.18	0.235^†^	1.98 ± 2.31	1.80 ± 2.30	0.658^†^
Margarine	2.00 ± 1.73	1.94 ± 1.61	0.963^‡^	0.53 ± 1.28	0.52 ± 1.38	0.985^‡^
Table sugar	4.40 ± 2.61	4.29 ± 2.77	0.932^‡^	3.14 ± 3.69	4.35 ± 5.60	0.766^‡^
Honey/jam	4.28 ± 2.17	3.78 ± 2.15	0.196^†^	4.79 ± 4.87	4.02 ± 5.65	0.184^‡^
Molasses	3.78 ± 2.31	3.17 ± 2.40	0.138^†^	4.05 ± 5.52	3.17 ± 5.46	0.094^‡^

Food consumption frequency codes (over the past month): never (1), once a month (2), 2–3 times a month (3), 1–2 times a week (4), 3–4 times a week (5), 5–6 times a week (6), once a day (7) and 2 or more a day (8)

†Independent samples t-test,

‡Mann–Whitney U test, *p* < 0.05*

The daily dietary intake and percentages of individuals meeting the requirements according to the DRIs of both groups are presented in Table 5. The control group had significantly higher dietary intake of carbohydrates, soluble fiber, insoluble fiber, total fiber, water, MUFAs, oleic acid, omega-3 fatty acids, folate, vitamin C, potassium, iodine, fluoride, and total minerals (*p* > 0.05). Compared with those in the control group, the dietary intake of cholesterol and vitamin D, and the omega-6/omega-3 fatty acid ratio were greater in the case group without statistical significance (*p* > 0.05). The mean percentage of meeting for protein and potassium was classified as “sufficient” for the control group, whereas “acceptable” for the case group, folate was classified as “acceptable” for the control group and “insufficient” for the case group. In addition, vitamin D, iron and fluoride were “insufficient” for both groups.

**Table 5:** Daily dietary energy and nutrient intake and percentages of individuals meeting the requirements according to the DRIs

	Daily intake		Meeting requirements (%) according to the DRIs (RDA or AI^a^)		
Variables	Control group (*n* = 65)	Case group (*n* = 65)	Control group (*n* = 65)	Case group (*n* = 65)	p
Energy (kcal)	1772.43 ± 746.01	1548.50 ± 628.40	n.a	n.a	0.061
Carbohydrates (g)	150.88 ± 71.19	126.01 ± 61.31	86.22 ± 40.68	72.01 ± 35.04	0.016*
Soluble fiber (g)	7.44 ± 3.76	5.87 ± 3.09	n.a	n.a	0.008*
Insoluble fiber (g)	14.72 ± 7.31	11.84 ± 6.24	n.a	n.a	0.011*
Total fiber (g)	25.05 ± 13.01	20.00 ± 10.52	89.45 ± 46.48^a^	71.42 ± 37.58^a^	0.011*
Water (ml)	1273.20 ± 533.39	1067.16 ± 541.88	n.a	n.a	0.008*
Fat (g)	95.84 ± 43.66	85.37 ± 38.97	n.a	n.a	0.109
Saturated fatty acids (g)	36.87 ± 18.87	32.84 ± 15.60	n.a	n.a	0.248
Monounstaturated fatty acids (g)	33.76 ± 14.52	29.39 ± 13.65	n.a	n.a	0.044*
Oleic acid (g)	29.68 ± 12.94	25.81 ± 12.48	n.a	n.a	0.037*
Polyunsaturated fatty acis (g)	19.24 ± 12.33	17.68 ± 12.92	n.a	n.a	0.127
Omega-3 fatty acids (g)	2.69 ± 1.31	2.22 ± 1.30	n.a	n.a	0.007*
Omega-6 fatty acids (g)	16.34 ± 11.18	15.27 ± 11.89	n.a	n.a	0.181
Omega-6/Omega-3	6.10 ± 2.26	6.94 ± 3.64	n.a	n.a	0.335
Cholesterol (mg)	358.75 ± 191.71	363.85 ± 190.03	n.a	n.a	0.889
Protein (g)	72.66 ± 29.67	66.24 ± 28.65	102.34 ± 41.79	93.30 ± 40.35	0.119
Essential amino acids (g)	34.06 ± 13.80	31.52 ± 13.82	n.a	n.a	0.167
Non-essential amino acids (g)	33.26 ± 13.68	30.36 ± 12.99	n.a	n.a	0.127
Vitamin A (Retinol) (µg)	642.48 ± 344.42	594.64 ± 282.49	83.44 ± 44.73	77.23 ± 36.69	0.666
Vitamin D (µg)	1.65 ± 1.31	1.78 ± 1.39	10.97 ± 8.74	11.87 ± 9.27	0.944
Vitamin E (mg)	17.59 ± 11.86	17.22 ± 12.23	117.27 ± 79.08	114.77 ± 81.55	0.591
Vitamin K (µg)	487.96 ± 278.08	429.59 ± 311.91	542.18 ± 308.98^a^	477.32 ± 346.57^a^	0.107
Thiamin (mg)	0.87 ± 0.54	0.76 ± 0.42	62.39 ± 38.71	54.11 ± 30.11	0.193
Riboflavin (mg)	1.74 ± 0.78	1.53 ± 0.75	124.57 ± 55.66	109.04 ± 53.24	0.070
Niacin (mg)	23.25 ± 9.26	21.39 ± 9.27	129.15 ± 51.46	118.83 ± 51.53	0.145
Pantothenic acid (mg)	5.75 ± 2.54	5.13 ± 2.28	95.85 ± 42.25^a^	85.46 ± 37.98^a^	0.085
Pyridoxine (mg)	1.42 ± 0.65	1.24 ± 0.56	74.92 ± 34.15	65.02 ± 29.72	0.050
Biotin (µg)	44.45 ± 18.55	39.78 ± 19.68	148.17 ± 61.84^a^	132.61 ± 65.59^a^	0.084
Folate (µg)	334.30 ± 144.07	290.91 ± 136.58	55.72 ± 24.01	48.48 ± 22.76	0.042^*^
Vitamin B12 (µg)	4.83 ± 2.66	4.31 ± 2.54	185.92 ± 102.24	165.61 ± 97.50	0.102
Vitamin C (mg)	159.18 ± 93.70	124.83 ± 80.15	198.98 ± 117.12	156.04 ± 100.19	0.026^*^
Calcium (mg)	1186.59 ± 561.80	1023.72 ± 518.69	118.66 ± 56.18	102.37 ± 51.87	0.061
Phosphorus (mg)	1407.17 ± 622.81	1259.35 ± 553.59	201.02 ± 88.97	179.91 ± 79.08	0.087
Sodium (mg)	2527.00 ± 1134.44	2262.92 ± 1308.88	168.47 ± 75.63^a^	150.86 ± 87.26^a^	0.058
Potassium (mg)	3087.21 ± 1369.36	2587.75 ± 1266.90	106.46 ± 47.22^a^	89.23 ± 43.69^a^	0.012^*^
Magnessium (mg)	340.56 ± 198.22	303.29 ± 166.15	97.30 ± 56.64	86.65 ± 47.47	0.113
Chloride (mg)	4066.03 ± 1829.45	3631.63 ± 2084.43	176.78 ± 79.54^a^	157.90 ± 90.63^a^	0.050
Sulfur (mg)	838.47 ± 328.82	760.47 ± 313.48	n.a	n.a	0.117
Iron (mg)	11.70 ± 5.86	10.22 ± 5.39	43.35 ± 21.72	37.88 ± 19.96	0.086
Zinc (mg)	9.97 ± 4.46	9.01 ± 3.83	90.60 ± 40.52	81.91 ± 34.85	0.121
Copper (mg)	1.78 ± 1.06	1.56 ± 0.88	177.64 ± 106.31	156.06 ± 87.85	0.100
Iodine (mg)	149.71 ± 71.39	133.34 ± 87.40	68.05 ± 32.45	60.61 ± 39.73	0.029^*^
Fluoride (µg)	580.16 ± 230.61	498.71 ± 242.02	19.34 ± 7.69^a^	16.62 ± 8.07^a^	0.020^*^
Manganese (mg)	3.47 ± 1.84	2.95 ± 1.62	173.37 ± 91.94^a^	147.71 ± 81.17^a^	0.056
Total minerals (mg)	17.89 ± 7.44	15.55 ± 7.34	n.a	n.a	0.019^*^

DRI values taken from Food and Nutrition Board, Institute of Medicine. DRI: Dietary reference intake. RDA: Recommended Dietary Allowance (shown in ordinary type). AI: Adequate intake (shown in ordinary type followed by an a (^a^)). n.a.: Not available. Mann–Whitney U test, *p* < 0.05*

Adjusted binary logistic regression analysis was conducted to further analyze the impact of the intake of various nutrients on the risk of EPL (Table 6). In all three models, higher dietary fiber intake as a continuous variable and quartile (Q3 and Q4) was inversely associated with the risk of EPL (*p* < 0.05). In Model 2, after adjusting for all potential confounders, compared with those in the reference quartile (Q1), participants in the third quartile (Q3) of dietary fiber intake had a 79.0% lower risk (OR = 0.21, 95% CI: 0.05–0.88, *p* = 0.032), and those in the fourth quartile (Q4) had an 80.0% lower risk (OR = 0.20, 95% CI: 0.05–0.82, *p* = 0.025) of EPL. Similarly, higher vitamin C intake, as a continuous and in the fourth quartile (Q4) was inversely associated with the risk of EPL (*p* < 0.05) in all three models. After adjusting for all potential confounders, compared with those in the reference quartile (Q1), participants in the fourth quartile (Q4) of vitamin C intake had an 83.0% lower risk (OR = 0.17, 95% CI: 0.04–0.77, *p* = 0.021) of EPL.

**Table 6:** Adjusted binary logistic regression analysis with EPL risk for the intake of various nutrients

	Crude Model		Model 1		Model 2	
	OR (95% CI)	p	OR (95% CI)	p	OR (95% CI)	p
Carbohydrates (g)						
Continuous	0.99 (0.99, 1.00)	0.042*	0.99 (0.99, 1.00)	0.041*	0.93 (0.99, 1.00)	0.074
Q1	Ref					
Q2	0.37 (0.13, 1.03)	0.057	0.35 (0.12, 1.00)	0.049	0.69 (0.17, 2.81)	0.601
Q3	0.29 (0.10, 0.81)	0.018*	0.28 (0.09, 0.79)	0.017*	0.36 (0.08, 1.62)	0.185
Q4	0.24 (0.08, 0.67)	0.007*	0.24 (0.08, 0.68)	0.007*	0.19 (0.05, 0.80)	0.023*
Total fiber (g)						
Continuous	0.96 (0.93, 0.99)	0.022*	0.96 (0.93, 0.99)	0.022*	0.95 (0.91, 0.99)	0.028*
Q1	Ref					
Q2	0.45 (0.18, 1.33)	0.159	0.49 (0.18, 1.35)	0.166	0.44 (0.11, 1.81)	0.255
Q3	0.34 (0.12, 0.93)	0.035*	0.34 (0.12, 0.96)	0.041*	0.21 (0.05, 0.88)	0.032*
Q4	0.27 (0.10, 0.77)	0.014*	0.27 (0.10, 0.77)	0.014*	0.20 (0.05, 0.82)	0.025*
Monounstaturated fatty acids (MUFA’s) (g)						
Continuous	0.98 (0.95, 1.00)	0.086	0.98 (0.95, 1.00)	0.084	0.99 (0.96, 1.02)	0.486
Q1	Ref					
Q2	0.92 (0.34, 2.51)	0.875	0.93 (0.34, 2.52)	0.880	1.61 (0.40, 6.48)	0.505
Q3	0.30 (0.11, 0.83)	0.020*	0.31 (0.11, 0.85)	0.023*	0.68 (0.18, 2.67)	0.585
Q4	0.47 (0.17, 1.27)	0.135	0.47 (0.17, 1.28)	0.138	0.66(0.16, 2.65)	0.554
Omega-3 (g)						
Continuous	0.75 (0.56, 0.99)	0.045*	0.74 (0.56, 0.99)	0.045*	0.84 (0.59, 1.19)	0.315
Q1	Ref					
Q2	0.37 (0.13, 1.03)	0.057	0.37 (0.13, 1.04)	0.058	0.53 (0.14, 2.08)	0.363
Q3	0.29 (0.10, 0.81)	0.018*	0.29 (0.10, 0.81)	0.018*	0.72 (0.19, 2.82)	0.640
Q4	0.24 (0.08, 0.67)	0.007*	0.23 (0.08, 0.67)	0.007*	0.53 (0.11, 1.78)	0.244
Folate (µg)						
Continuous	0.99 (0.99, 1.00)	0.086	0.99 (0.99, 1.00)	0.088	0.99 (0.99, 1.00)	0.121
Q1	Ref					
Q2	0.64 (0.24, 1.71)	0.372	0.64 (0.24, 1.72)	0.374	0.85 (0.22, 3.36)	0.818
Q3	0.64 (0.24, 1.71)	0.372	0.64 (0.24, 1.73)	0.381	0.73 (0.20, 2.71)	0.635
Q4	0.31 (0.11, 0.87)	0.026*	0.32 (0.11, 0.89)	0.029*	0.29 (0.07, 1.15)	0.079
Vitamin C (mg)						
Continuous	0.99 (0.99, 1.00)	0.031*	0.99 (0.99, 1.00)	0.031*	0.99 (0.99, 1.00)	0.022*
Q1	Ref					
Q2	0.64 (0.24, 1.71)	0.372	0.64 (0.24, 1.73)	0.381	0.91 (0.23, 3.59)	0.898
Q3	0.64 (0.24, 1.71)	0.372	0.65 (0.24, 1.75)	0.391	0.48 (0.11, 2.02)	0.317
Q4	0.31 (0.11, 0.87)	0.026*	0.32 (0.11, 0.89)	0.029*	0.17 (0.04, 0.77)	0.021*

*p* < 0.05*

The fourth quartile (Q4) of carbohydrates intake was negatively associated with the risk of EPL in all three models compared with the reference quartile (Q1) (*p* < 0.05). Additionally, higher omega-3 fatty acid intake as a continuous variable and in the third (Q3) and fourth (Q4) quartile were inversely associated with the risk of EPL (*p* < 0.05) in both the crude model and Model 1.

## Discussion

In this study, we aimed to evaluate the first trimester diet and nutritional knowledge level of women who had EPL and to compare them with those of healthy pregnant women after controlling for other EPL risk factors. We have shown that notable differences in nutritional and meal behaviors along with daily nutrient intake, despite the nutritional knowledge levels were similar in both groups.

Dietary fiber is an edible plant-based carbohydrate polymers that are partially or completely digested and absorbed in the colon [36] and may play an intriguing role in the relationship between maternal microbiome and obstetric outcomes [37]. Adequate dietary fiber intake during pregnancy is associated with improved insulin sensitivity [36], microbial diversity in the gut and vaginal microbiota and enhanced intestinal barriers [37], and improved pregnancy outcomes [38]. In a systematic review and meta-analysis study, high fiber diet was associated with decreased risk of gestational DM and hypertensive disorders [39]. In a randomized controlled trial, Shen et al. (2024) demonstrated that, although not associated with pregnancy outcomes, daily soluble dietary fiber supplementation is a safe and tolerable intervention that reduces the need of constipation medication during pregnancy [36]. According to a systematic review, adequate intake of dietary fiber during pregnancy has been suggested to provide protective effects against PL through various mechanisms [40]. In other study, Ahmadi et al. (2017) reported that the mean daily consumption of high fiber foods, including vegetables and fruits was lower in the EPL group compared with the healthy pregnant women, however the total dietary fiber intake of the groups were not reported [41]. Conversely, Willis et al. (2022) found that there was no association between dietary fiber intake during the preconception period and PL [42]. In our study, the primary finding was the soluble, insoluble and total dietary fiber intakes were higher in the control group. In addition, the results of the adjusted binary logistic regression analysis demonstrated that higher total dietary fiber intake (as a continuous variable) and in the third (Q3) and fourth (Q4) quartile were associated with a decreased risk of EPL. Even after adjusting for potential confounders for the risk of EPL, the inverse association remained significant.

Having regular meals during the day may support circadian rhythm and metabolic regulation [43]. It has been reported that consuming a greater number of meals was associated with increased general dietary quality and a reduced risk of pregnancy complications [44, 45]. While previous studies have focused on the link between meal behaviors and other pregnancy outcomes including poor sleep and emotion [46], preterm delivery [47] and neonatal anthropometric measurements [48], the risk of EPL has not been adequately addressed. In our study, we found that the frequency of eating between meals and the mean total number of daily meals were higher in the control group.

During pregnancy, the requirements of nearly all macro and micronutrients increase to meet the physiological needs of both the mother and the fetus [34]. The diet is rich in vitamins and minerals and contributes to healthy pregnancy [30]. We found that dietary folate, vitamin C, potassium, iodine, fluoride, water, and total minerals intake were higher in the control group than in the case group. These findings are in concordance with previous studies reporting an inverse association between folate intake [49, 50], vitamin C intake [9, 41, 51, 52], maternal serum potassium level [53, 54] and PL risk.

The World Health Organization (WHO) recommends the use of dietary supplements, especially when dietary intake is insufficient, during pregnancy to meet increased nutrient requirements [55]. In a comprehensive systematic review, it has been concluded that while there is no evidence to support the use of single-vitamin supplementation, multivitamin supplementation may reduce the risk of PL by improving maternal nutritional status [56]. A study conducted among pregnant women with a history of recurrent EPL, demonstrated that first trimester antioxidant supplementation (zinc, selenium, vitamin c and vitamin E) together with folic acid improved the ongoing pregnancy rate compared to the placebo group (folic acid supplementation only) [57]. In the present study, dietary supplement use upon medical advice were more common in the control group. These findings are consistent with previous studies reporting that vitamin supplement use was more common in the healthy pregnant women compared with PL group [58, 59]. However, Zhang et al. (2022) observed that multivitamin supplementation was more prevalent in the PL group both during the preconception period and/or in the first trimester [9].

The literature indicates that adequate carbohydrate intake is essential for tolerating maternal physiological changes [60] and meeting maternal and fetal energy requirements and the development of fetal tissues [61, 62]. In a study by Willis et al. (2022), a high carbohydrate diet during the preconception period was not associated with the risk of PL [42]. In contrast, our study revealed that daily dietary carbohydrate intake and the average percentage of meeting total carbohydrate requirements were higher in the control group in the first trimester. Additionally, according to the results of the adjusted binary logistic regression analysis, the highest quartile (Q4) of carbohydrate intake was negatively associated with the risk of EPL in all three models compared with the reference quartile (Q1).

Omega-3 fatty acids reportedly support reproductive organ function [63], and supplementation with omega-3 fatty acids during pregnancy may reduce the risk of recurrent PL by reducing oxidative stress [64]. Additionally, a dietary omega-6/omega-3 ratio of 1:1 to 2:1 is recommended to promote general health [65]. In one study, Li et al. (2018), reported that the arachidonic acid (a type of omega-6 fatty acid)/eicosapentaenoic acid (an omega-3 fatty acid) ratio in placental villi was significantly higher in the EPL group than in healthy pregnant women who requested legal induced abortion in the first trimester [66]. We observed that dietary omega-3 fatty acid intake was higher in the control group, and the omega-6/omega-3 fatty acid ratio was higher in the case group. In this study, adjusted binary logistic regression analysis data suggest that higher omega-3 fatty acid intake as a continuous variable and in third (Q3) and fourth (Q4) quartile decrease EPL risk in two different models.

Improved nutrition knowledge level may contribute to greater adherence to healthy eating behaviors during pregnancy. However, factors such as lower household income and limited access to healthy food may limit act as a barrier to healthy eating, even in individuals with high levels of nutritional education [67]. In the present study, considering participants’ nutrition knowledge levels, according to the NKLSA scoring criteria both groups were classified as moderate in the “Basic nutrition” section. And regarding the “Food choices” section, the scores were classified as high in control group while moderate in the case group. Our findings support recent studies showing that the nutritional knowledge of pregnant women in Turkey is at a moderate to high levels, as assessed by NKLSA [68, 69]. In our study, difference between the mean scores for these two sections which directly assess nutritional knowledge level was not statistically significant. Despite similar nutrition knowledge levels, knowledge-behavior gap, socioeconomic differences and motivational factors may be potential explanations for the observed differences in dietary behaviors. Also, higher VAS scores observed in the control group on the “Subjective assessment of the nutrition-health relationship” sub-item of the NKLSA may be associated with a greater translation of nutritional knowledge into healthy eating behaviors.

### Strengths and limitations

The primary limitations of this study include the small sample size because nearly one out of two participants were excluded based on the strict eligibility criteria (Fig. 1). Furthermore, there was a difference in gestational age between the two groups despite all participants were in the first trimester. This difference may be attributed to the delayed awareness of pregnancy [70] and hospital visit time for the healthy pregnant group [71] and the occurrence of EPL mostly between the 6 and 8 weeks, and early hospital visit time for the case group [72]. Despite this difference between the two groups, evidence indicates that energy and nutrient requirements and intakes increase mainly after the first trimester; therefore, the two groups’ nutrient requirements and intakes are likely comparable [73, 74]. We also acknowledge that participants’ dietary intake was assessed via the QFFQ instead of three-day food records, which could provide detailed information regarding dietary patterns, due to limited time. And only foods from the Turkish Dietary Guidelines were examined, while fast food or packaged food consumption was not investigated. In addition, information about the distribution of daily energy intake according to the time of day, appetite status and gastrointestinal symptoms were not collected. These limitations may be insufficient to reflect the overall nutrient intake or eating habits of participants. Despite its limitations, this study has several notable strengths. Firstly, we applied strict exclusion criteria to minimize non-dietary risk factors and specifically examine the impact of maternal diet on EPL. Moreover, by recruiting all participants from the same hospital and catchment area, we aimed to control the effect of environmental and sociodemographic factors. Another strength include data collection was performed by a qualified dietitian, the use of visual materials to improve the accuracy for estimation of portion size estimation. Finally, we employed binary logistic regression models to adjust for potential cofounders, which enabled a reliable evaluation of the independent effect of specific nutrient. To the best of our knowledge this study is among the limited number of studies to investigate the effects of nutritional variables in detail and together with EPL after controlling other risk factors.

## Conclusions

In conclusion, this study contributes to the literature by demonstrating notable differences in nutritional and meal behaviors between healthy pregnant women and women who had EPL in the first trimester despite their similar nutritional knowledge levels. Although eating habits and insufficient nutrient intake may not be the sole determinant of EPL, improving overall diet quality including adequate dietary fiber intake may contribute to reducing the risk of EPL. Considering that maternal nutritional status during the first trimester is a modifiable risk factor, it should be prioritized in public health interventions aimed at preventing EPL. Further studies examining an in-depth assessment of the dietary patterns of the groups in a larger sample are needed to better understand the role of first trimester maternal nutritional status in EPL risk.

## Data Availability

The datasets used and/or analyzed during the current study are available from the corresponding author on reasonable request.

## Abbreviations

ACOG: American College of Obstetricians and Gynecologists
AI: Adequate intake
BEBIS: Nutrition Information System
BMI: Body mass index
DM: Diabetes mellitus
DRI: Dietary Reference Intakes
EPL: Early pregnancy loss
FFQ: Food Frequency Questionnaire
ICD-10: International Classification of Diseases, Tenth Revision
MUFAs: Monounsaturated fatty acids
NKLSA: Nutrition Knowledge Level Scale for Adults
PL: Pregnancy loss
QFFQ: Quantitative Food Frequency Questionnaire
RDA: Recommended Dietary Allowance
TUBER: Turkey Dietary Guidelines
VAS: Visual Analogue Scale
